# Association between obstructive sleep apnea and visceral adiposity index and lipid accumulation product: NHANES 2015–2018

**DOI:** 10.1186/s12944-024-02081-5

**Published:** 2024-04-10

**Authors:** Tingfeng Zhou, Shihao Chen, Jiesheng Mao, Pei Zhu, Xinru Yu, Renyu Lin

**Affiliations:** 1 Department of Otolaryngology, The First Affiliated Hospital of Wenzhou Medical University, Wenzhou, China; 2 Department of Neurology, The First Affiliated Hospital of Wenzhou Medical University, Wenzhou, China; 3 Department of Neurology, Postgraduate Training Base Alliance of Wenzhou Medical University (WenzhouPeople’s Hospital), Wenzhou, China; 4 Department of Ophthalmology, The First Affiliated Hospital of Wenzhou Medical University, Wenzhou, China

**Keywords:** Obstructive sleep apnea, Visceral adiposity index, Lipid accumulation product, NHANES, Abdominal obesity

## Abstract

**Background:**

Obesity refers to a significant contributor to the development of obstructive sleep apnea (OSA). Early prediction of OSA usually leads to better treatment outcomes, and this study aims to employ novel metabolic markers, visceral adiposity index (VAI), and lipid accumulation product (LAP) to evaluate the relationship to OSA.

**Methods:**

The data used in the current cross-sectional investigation are from the National Health and Nutrition Examination Survey (NHANES), which was carried out between 2015 and 2018. To examine the correlation between LAP and VAI levels and OSA, multivariate logistic regression analysis was adopted. In addition, various analytical methods were applied, including subgroup analysis, smooth curve fitting, and threshold effect analysis.

**Results:**

Among totally 3932 participants, 1934 were included in the OSA group. The median (Q1-Q3) values of LAP and VAI for the participants were 40.25 (21.51–68.26) and 1.27 (0.75–2.21), respectively. Logistic regression studies indicated a positive correlation between LAP, VAI, and OSA risk after adjusting for potential confounding variables. Subgroup analysis revealed a stronger correlation between LAP, VAI levels, and OSA among individuals aged < 60 years. Through smooth curve fitting, specific saturation effects of LAP, VAI, and BMD were identified, with inflection points at 65.684 and 0.428, respectively.

**Conclusion:**

This study demonstrates that elevated levels of LAP and VAI increase the risk of OSA, suggesting their potential as predictive markers for OSA and advocating for dietary and exercise interventions to mitigate OSA risk in individuals with high LAP and VAI levels.

## Introduction

The hallmark of obstructive sleep apnea (OSA) is the partial or total collapse of the upper airway for at least ten seconds while you are asleep, resulting in reduced airflow (hypoventilation) or complete cessation (apnea). Its primary symptom is excessive daytime drowsiness [1, 2]. Based on epidemiological studies, OSA influences approximately 17% of women and 34% of men aged between 30 and 70 in the United States [3]. Left untreated, OSA can lead to severe health complications, including hypertension [4], cardiovascular diseases [5], and diabetes [6]. Therefore, it is crucial to find new and more accurate biomarkers for the early diagnosis of OSA is crucial.

Recently, OSA has been regarded to be one of the serious complications caused by obesity [7]. Obesity is characterized by the accumulation of visceral fat. However, conventional measures, including BMI, can only roughly assess obesity status and cannot differentiate between subcutaneous and visceral fat. As a novel anthropometric index, the visceral adiposity index (VAI) has been shown to be effective in evaluating adult visceral fat distribution and dysfunction. Waist circumference (WC) and body mass index(BMI) can be applied to compute VAI, which can additionally evaluate triglyceride (TG) and high-density lipoprotein (HDL) [8]. Clinical studies have shown that VAI can effectively identify individuals at higher risk of metabolic disorders associated with visceral obesity, including insulin resistance, lipid abnormalities, and cardiovascular risk factors [9–11]. In addition, the lipid accumulation product (LAP) index, derived from the combination of waist circumference and TG, can also assess and show the status of abdominal lipid accumulation. Its predictive efficacy is superior to traditional indicators like BMI and waist-height ratio (WHtR) [12, 13], and it has also exhibited good predictive performance in some clinical conditions [14, 15].

The National Health and Nutrition Examination Survey (NHANES) is a meticulously designed large-scale clinical registry database with complete follow-up, making it well-suited for discussing the relationship between VAI and LAP with OSA. Thus, this study is aimed at evaluating the association between VAI and LAP for OSA, and we assume that VAI and LAP make a strong predictive effect on OSA. This study comprehensively evaluates OSA using VAI and LAP through effectively utilizing the powerful advantages of the NHANES database and expects the research results to provide guiding significance for clinical practice.

## Methods

### Study population and research design

NHANES, a nationally representative, stratified, multistage probability sample survey, is performed by the National Center for Health Statistics (NCHS) [16]. Its aim is to evaluate the relationship between nutrition health promotion and illness prevention. This survey combines interviews with physical examinations and is performed in two-year cycles, covering demographic, dietary, examination, laboratory, and questionnaire sections. More information concerning the NHANES database can be accessed at http://www.cdc.gov/nhanes.

**Fig. 1:**
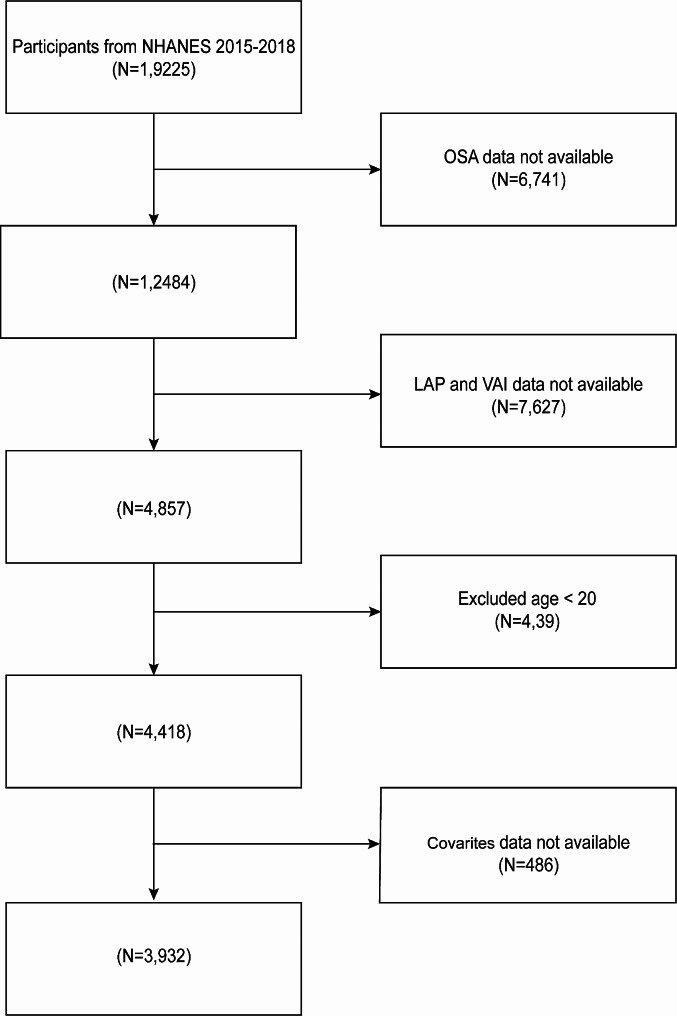
Flow chart of patient screening

Between 2015 and 2018, totally 19,225 participants were involved. Based on strict inclusion and exclusion criteria, this study ultimately determined a sample size of 3932 U.S. adults from the NHANES 2005–2018 cycle. Specifically, this study excluded 6741 participants with missing OSA data, 7627 participants with missing VAI and LAP data, 439 individuals under 20 years of age, and 486 participants with missing covariate values (Fig. 1).

### Assessment of OSA

In accordance with earlier studies, OSA is diagnosed when a person answers “yes” to at least one of the following three NHANES questions [17]: (1) being excessively sleepy in the day even though they get at least 7 h of sleep per night, as reported 16–30 times; (2) experiencing episodes of gasping, snorting, or stopping their breath on three or more occasions per week; (3) snoring on three or more occasions every week.

### Assessment of LAP and VAI

Based on previously established equations indicated by Kahn and Amato et al. [12, 18], LAP and VAI were calculated according to gender using the following formulas, where TG and HDL are in mmol/L, WC is in cm, and BMI is in kg/m^2.

$$Males:LAP=\left(WC-65\right)*TG$$

$$Females:LAP=\left(WC-58\right)*TG$$

$$Males:VAI=\left(\frac{\text{W}\text{C}}{39.68+1.88\text{*}\text{B}\text{M}\text{I}}\right)*\left(\frac{\text{T}\text{G}}{1.03}\right)*\left(\frac{1.31}{\text{H}\text{D}\text{L}}\right)$$

$$Females:VAI=\left(\frac{\text{W}\text{C}}{36.58+1.89\text{*}\text{B}\text{M}\text{I}}\right)*\left(\frac{\text{T}\text{G}}{0.81}\right)*\left(\frac{1.52}{\text{H}\text{D}\text{L}}\right)$$

### Measurement of covariates

Based on previous studies [19, 20], potential confounding factors related to VAI, LAP, and OSA were included in the final analysis. Age, race, gender, educational attainment, marital status, and the income-to-poverty ratio (PIR) were among the demographic factors. Mexican Americans, non-Hispanic whites, non-Hispanic blacks, other Hispanics, and other races were shown to be the different categories for race. Educational level was divided into the following three types: below high school, high school or above, and above high school. Marital status was categorized into two types: “married or partnered” and “living alone”. BMI, calculated through dividing weight (in kilograms) by height (in meters squared), was also regarded as an examination variable. Questionnaire surveys included alcohol consumption, smoking status, and diabetes. Participants self-reported their medical history to assess chronic health conditions, indicating whether a doctor or other health professional had informed them of specific health conditions. Participants’ smoking status was categorized in line with whether or not they had ever smoked 100 cigarettes or more. “Ever have 4/5 or more drinks every day” was the definition of alcohol consumption status.

### Statistical analysis

VAI and LAP were divided into quartiles (Q1 to Q4) from lowest to highest. Categorical characteristics were reported as proportions, whereas continuous variables were summarized as means with standard errors (SE). Weighted Student’s t-tests or weighted chi-square tests were utilized to explore differences between individuals stratified by gender for continuous variables.

For the purpose of exploring the odds ratios (ORs) and 95% confidence intervals (CIs) between OSA and LAP, VAI, multiple logistic regression models were adopted. Three models were applied: Model 1 (unadjusted), Model 2 (adjusted for age, gender, and race only), and Model 3 (fully adjusted for age, race, gender, educational level, income-to-poverty ratio, marital status, BMI, alcohol consumption, smoking status, and diabetes). Interaction and subgroup analyses were performed to explore potential modifications to this association by covariates.

Additionally, smooth curve fitting based on generalized additive models (GAM) was used to evaluate the nonlinear relationship between VAI, LAP, and OSA.

*P* < 0.05 was applied as the statistical significance threshold. R (version 3.4.3) and EmpowerStats (version 2.0) were adopted for the statistical analysis.

## Results

### Baseline characteristics of participants

Totally 3932 individuals met the study criteria and were involved in the analysis. Table 1 outlines the baseline characteristics of participants grouped by the presence or absence of OSA, with 1934 individuals being included in the OSA group. The mean age of participants was 50.21 ± 17.32 years, with females comprising 51.32% and males 48.68%. The Median (Q1-Q3) of LAP and VAI were 40.25 (21.51–68.26) and 1.27 (0.75–2.21), respectively. Participants in the OSA group had higher VAI and LAP values when compared with those in the non-OSA group, with statistical significance. In addition, significant differences were observed among all OSA patients in terms of age, gender, race, marital status, educational level, alcohol consumption history, and diabetes history (*P* < 0.05).

**Table 1:** Baseline characteristics of participants in the NHANES 2015–2018

Characteristics	Overall	OSA(*N* = 1934)	Non-OSA(*N* = 1934)	*P*-value
**Continuous variables, mean ± SD**				
Age (years)	50.21 ± 17.32	51.66 ± 16.25	48.79 ± 18.18	< 0.0001
BMI (kg/m2)	29.58 ± 7.09	31.11 ± 7.26	28.10 ± 6.60	< 0.0001
Waist Circumference (cm)	100.58 ± 16.90	104.49 ± 16.65	96.79 ± 16.27	< 0.0001
Income to poverty ratio	2.52 ± 1.60	2.50 ± 1.59	2.53 ± 1.61	0.630
Total Cholesterol (mmol/L)	1.29 ± 1.12	1.40 ± 1.30	1.18 ± 0.89	< 0.0001
HDL-C (mmol/L)	1.41 ± 0.43	1.35 ± 0.40	1.46 ± 0.45	< 0.0001
LAP, Median (Q1-Q3)	40.25 (21.51–68.26)	48.38 (27.51–78.48)	33.97 (17.36–57.57)	< 0.0001
VAI,Median (Q1-Q3)	1.27 (0.75–2.21)	1.43 (0.86–2.43)	1.15 (0.68–1.96)	< 0.0001
**Categorical variables,%**				
**Gender**				< 0.0001
Male	48.68	52.79	44.69	
Female	51.32	47.21	55.31	
**Race**				0.0190
Mexican American	14.93	16.80	13.11	
Other Hispanic	11.57	11.84	11.31	
Non-Hispanic White	35.48	34.59	36.34	
Non-Hispanic Black	20.80	20.32	21.27	
Other Races	17.22	16.44	17.97	
**Education level**				0.044
Less than high school	43.23	44.21	42.29	
High school or GED	31.74	32.52	30.98	
Above high school	35.33	23.27	26.73	
**Marital status**				
Married/Living with a partner	60.89	64.99	56.91	< 0.0001
Living alone	39.11	35.01	43.09	
**Alcohol drinking**				0.065
Yes	17.08	18.28	15.85	
No	82.92	81.72	84.15	
**Smoked at least 100 cigarettes**				< 0.0001
Yes	44.05	48.19	40.04	
No	55.95	51.81	59.96	
**Diabetes**				< 0.0001
Yes	15.82	18.77	12.96	
No	81.43	78.13	84.63	
borderline	2.75	3.10	2.40	

Abbreviations: OSA: Obstructive sleep apnea.; VAI: Visceral adiposity index; LAP: Lipid accumulation product; NHANES: National Health and Nutrition Examination Survey; SE: standard error

### Association between LAP, VAI, and OSA

With the purpose of further investigating the correlation between OSA and LAP, VAI, three multiple regression models were constructed for analysis (Table 2). In the unadjusted Model 1, multiple logistic regression analysis suggested that there existed a significant positive correlation between OSA and LAP, VAI. After the adjustment for all covariates in Model 3, the significant association between OSA and LAP, VAI persisted (OR = 1.002, 95% CI: 1.001, 1.004, *P* = 0.0069; OR = 1.042, 95% CI: 1.006, 1.080, *P* = 0.0219), suggesting that for every one-unit increase in LAP, VAI levels, the risk of OSA elevated by 0.2% and 4.2%, respectively. Subsequently, LAP and VAI were converted into categorical variables based on quartiles for sensitivity analysis. In the fully adjusted Model 3, when comparison with the lowest quartile, participants in the highest LAP and VAI quartiles exhibited a significantly increased risk of OSA by 45.5% and 29.5%, respectively, with statistical significance (OR = 1.455, 95% CI: 1.142, 1.853, *P* = 0.0024; OR = 1.295, 95% CI: 1.060, 1.583, *P* = 0.0115).

**Table 2:** Multivariable logistic regression models for the association between LAP、VAI, and OSA in adults in the NHANES 2015–2018

OSA	OR (95% CI), *P*-value		
	Model 1	Model 2	Model 3
**LAP**			
Continuous	1.008 (1.006, 1.009) < 0.0001	1.007 (1.006, 1.009) < 0.0001	1.002 (1.001, 1.004) 0.0068
Categories			
Q 1	Reference	Reference	Reference
Q 2	1.643 (1.370, 1.970) < 0.0001	1.567 (1.302, 1.887) < 0.0001	1.184 (0.972, 1.443) 0.0936
Q 3	2.277 (1.899, 2.731) < 0.0001	2.157 (1.788, 2.603) < 0.0001	1.353 (1.093, 1.676) 0.0055
Q 4	2.963 (2.466, 3.560) < 0.0001	2.862 (2.364, 3.464) < 0.0001	1.455 (1.142, 1.853) 0.0024
P for trend	< 0.0001	< 0.0001	0.0051
**VAI**			
Continuous	1.109 (1.068, 1.152) < 0.00001	1.103 (1.062, 1.146) < 0.00001	1.042 (1.006, 1.080) 0.0219
Categories			
Q 1	Reference	Reference	Reference
Q 2	1.343 (1.123, 1.606) 0.0012	1.313 (1.095, 1.573) 0.0032	1.092 (0.905, 1.318) 0.3574
Q 3	1.736 (1.451, 2.076) < 0.0001	1.686 (1.404, 2.026) < 0.0001	1.218 (1.003, 1.480) 0.0467
Q 4	1.971 (1.648, 2.359) < 0.0001	1.258 (1.177, 1.343) < 0.0001	1.295 (1.060, 1.583) 0.0115
P for trend	< 0.0001	< 0.0001	0.0121

Abbreviations: OSA: Obstructive sleep apnea.; VAI: Visceral adiposity index; LAP: Lipid accumulation product; NHANES: National Health and Nutrition Examination Survey; OR: odds ratio: CI: confidence interval

**Fig. 2:**
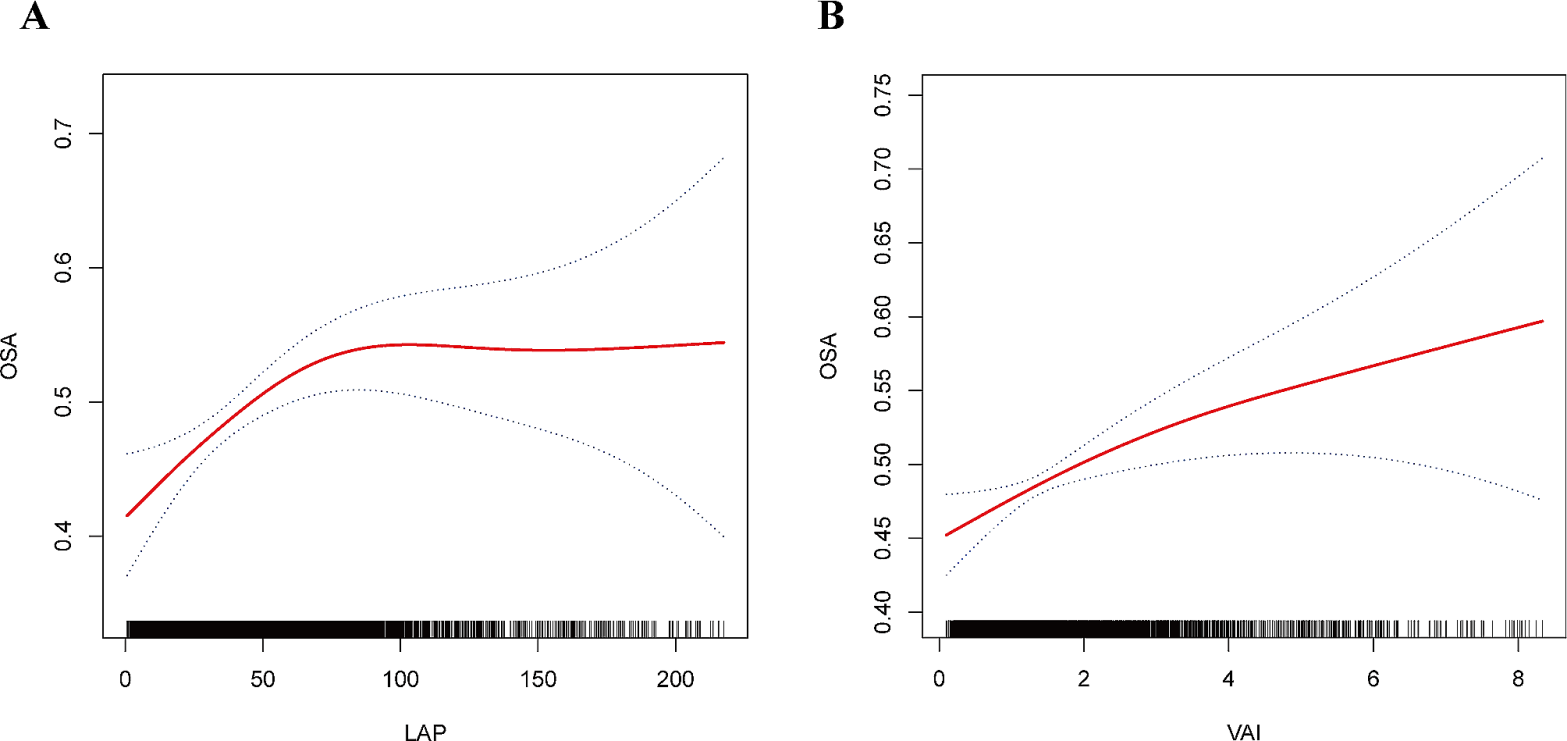
The nonlinear associations between the LAP (**A**, VAI (**B**) and OSA. The solid red line represents the smooth curve fit between variables. Blue bands represent the 95% confidence interval from the fit

To further visualize the correlation between LAP, VAI, and OSA, smooth curve fitting based on Model 3 was conducted. Results in Fig. 2 indicated a nonlinear relationship between LAP, VAI, and OSA. Then, threshold effect analysis was performed to clarify their relationship(Table 3). The inflection point for LAP was determined as 65.684 (log-likelihood ratio = 0.003), indicating that when LAP levels were below 65.684, every one-unit increase in LAP showed a relationship to a 0.9% elevation in the risk of OSA. When LAP levels exceeded 65.684, the correlation between LAP and OSA risk disappeared, suggesting that further increases in LAP did not significantly elevate the risk of OSA. Similar results were observed for VAI with an inflection point at 0.428 (log-likelihood ratio = 0.008), suggesting different effects of VAI on OSA risk below and above this threshold.

**Table 3:** Threshold effect analysis of LAP and VAI on OSA using a two-piecewise logistic regression model in adults in the NHANES 2015–2018

Threshold effect analysis	OSA OR (95%CI) *P*-value
**LAP**	
Inflection point of LAP (K)	65.684
<K slope	1.009 (1.004, 1.013) 0.0001
>K slope	0.999 (0.996, 1.002) 0.6561
Log-likelihood ratio test	0.003
**VAI**	
Inflection point of VAI (K)	0.428
<K slope	19.396 (2.155, 174.605) 0.0082
>K slope	1.069 (1.013, 1.128) 0.0151
Log-likelihood ratio test	0.008

Abbreviations: OSA: Obstructive sleep apnea.; LAP: Lipid accumulation product; NHANES: National Health and Nutrition Examination Survey;

Notes: Age, race, gender, level of education, marital status, poverty income ratio, body mass index, alcohol consumption, smoking status, and diabetes were adjusted

### Subgroup analyses

Multiple subgroup analyses and interaction tests based on various covariates were adopted for evaluating the robustness of the relationship between LAP, VAI, and OSA, and for identifying potential population differences (Table 4). The findings of subgroup analysis indicated a consistent relationship between LAP, VAI, and OSA across most subgroups. Notably, a significant interaction was observed between LAP, VAI, and age (interaction *P* < 0.05). In participants who aged < 60 years, a strong and significant positive correlation was observed (OR = 1.004, 95% CI: 1.002, 1.006, *P* = 0.00156; OR = 1.065, 95% CI: 1.020, 1.113, *P* = 0.0215), while this association became non-significant in participants aged ≥ 60 years. This suggests that higher levels of LAP and VAI may increase the likelihood of developing OSA in individuals aged < 60 years.

**Table 4:** Stratified analysis of the correlation between LAP、VAI and OSA in adults in the NHANES 2015–2018

Subgroup	OR (95% CI), *P*-value			
	LAP	P interaction	VAI	P interaction
**Age**		**0.0156**		**0.0215**
< 60	1.004 (1.002, 1.006) 0.0008		1.065 (1.020, 1.113) 0.0046	
> =60	0.999 (0.996, 1.002) 0.5545		0.970 (0.906, 1.039) 0.3841	
**Gender**		0.7949		0.7768
Male	1.002 (1.000, 1.005) 0.0318		1.035 (0.990, 1.082) 0.1277	
Female	1.002 (0.999, 1.005) 0.1674		1.046 (0.990, 1.106) 0.1119	
**BMI**		0.5129		0.4957
< 25	1.000 (0.993, 1.008) 0.9108		1.010 (0.886, 1.153) 0.8785	
25–30	1.001 (0.998, 1.004) 0.6419		1.013 (0.959, 1.070) 0.6354	
> 30	1.003 (1.001, 1.005) 0.0107		1.058 (1.003, 1.116) 0.0374	
**Diabetes**		0.3902		0.2707
Yes	1.002 (0.999, 1.005) 0.1715		1.026 (0.961, 1.096) 0.4350	
No	1.002 (1.000, 1.004) 0.0230		1.046 (1.003, 1.091) 0.0340	
Borderline	1.017 (0.995, 1.040) 0.1253		1.615 (0.910, 2.867) 0.1013	
**Smoke**		0.5284		0.3934
Yes	1.003 (1.000, 1.005) 0.0205		1.058 (1.005, 1.114) 0.0322	
No	1.002 (1.000, 1.004) 0.1143		1.026 (0.979, 1.076) 0.2842	
**Education level**		0.6183		0.4624
High school or less	1.001 (0.999, 1.004) 0.2685		1.017 (0.964, 1.074) 0.5338	
Some college or AA	1.003 (1.000, 1.006) 0.0257		1.069 (1.002, 1.140) 0.0423	
College graduate or above	1.003 (0.999, 1.007) 0.1168		1.058 (0.979, 1.143) 0.1558	

Abbreviations: OSA: Obstructive sleep apnea.; VAI: Visceral adiposity index; LAP: Lipid accumulation product; NHANES: National Health and Nutrition Examination Survey; OR: odds ratio: CI: confidence interval

Notes: Age, race, gender, level of education, marital status, poverty income ratio, body mass index, alcohol consumption, smoking status, and diabetes were adjusted. The strata variable was not included in the model when stratifying by itself

## Discussion

In the present cross-sectional study involving 3932 representative adults, this work discovered a significant positive correlation between VAI, LAP, and OSA. Importantly, non-linear relationships were observed between LAP, VAI, and OSA, with saturation values of LAP (65.684) and inflection points of VAI (0.428) identified for all participants. This suggests that there exists a strong correlation between LAP, VAI levels, and OSA within specific ranges, indicating the potential clinical significance of maintaining ideal LAP and VAI levels in lowering the risk of OSA.

Obstructive sleep apnea and obesity exhibit a complex interdependence [21]. Obesity, especially excess abdominal fat, is a main risk factor for the development and exacerbation of OSA. The accumulation of fat around the neck and upper airway can result in airway narrowing or obstruction during sleep, causing episodes of complete or partial cessation of breathing (apneas or hypopneas) [22, 23]. Abdominal obesity not only increases intra-abdominal pressure while lowers lung volume and increases the likelihood of upper airway collapse [24]. , but is also correlated with an increase in visceral fat, which in turn secretes various inflammatory and adipose factors, resulting in systemic inflammation and oxidative stress. This influences muscle activity in the upper airway and promotes the proliferation of adipose tissue around the upper airway, consequently increasing the risk of OSA [25, 26]. By contrast, OSA can also contribute to weight gain and difficulty in losing weight. Hormonal regulation may be disturbed by the irregular sleep patterns and frequent awakenings linked to OSA, which can result in an increase in hunger and desire for high-calorie foods [27–29]. In addition, daytime fatigue and reduced energy levels can decrease motivation for physical activity, further promoting weight gain [30]. Moreover, evidence suggests that OSA itself can change the lipid profile [31]; OSA itself exacerbates lipid abnormalities by increasing insulin resistance and inflammatory responses, creating a negative feedback loop [32, 33]. The elevation in blood lipids puts pressure on the cardiovascular system, elevating the workload of the heart and blood vessels, which is unfavorable for patients with OSA, as they are already under the cardiovascular burden caused by intermittent hypoxia. This bidirectional relationship between OSA and obesity creates a vicious cycle where one condition exacerbates the other. Both conditions share common risk factors, including a sedentary lifestyle, poor dietary habits, and genetic predisposition, which further complicates their relationship [34].

Compared with traditional methods, which include computed tomography and magnetic resonance imaging, VAI and LAP offer simple and non-invasive means to assess visceral fat accumulation. Introduced in 2010, VAI utilizes multiple metabolic markers to accurately assess visceral fat distribution and associated metabolic abnormalities. LAP, proposed in 2005, has been found to better predict cardiovascular diseases than traditional BMI [35, 36]. These indices provide additional tools for evaluating risk factors associated with obesity and metabolic abnormalities. A lot of studies have demonstrated the predictive capabilities of VAI and LAP in various diseases, including diabetes and cardiovascular diseases [37, 38].

This study reveals a positive association between VAI, LAP, and OSA, consistent with findings by Deng et al. Hence, both indices should be considered in clinical OSA management [21]. “Previous meta-analyses have also reported the similar findings [39], suggesting that lipid indexes, including LAP and VAI, possess commendable diagnostic capability ies. For the research findings, this study compared and discussed them with relevant literature. Firstly, both VAI and LAP have been found to predict cardiovascular diseases better when compared with individual lipid components and are both associated with OSA, a correlation the results also affirm. However, when comparing ROC curves, it could be found that the association between LAP and OSA is significantly stronger. LAP has also been identified as one of the best parameters for predicting hypertension, diabetes, and ischemic heart disease in OSA patients [40, 41]. In other studies, VAI has been identified as a marker for predicting insulin resistance in OSA patients [42], potentially providing a powerful tool for identifying OSA patients at risk of MetS [43]. In other study, LAP and CMI (cardiometabolic index) were shown to be related to OSA and MetS, but CMI has a more practical threshold in identifying disease states [44]. In the final study, LAP and the TyG index were indicated to be associated with an elevated risk of OSA and cardiometabolic diseases [45]. In summary, both VAI and LAP are related to OSA or cardiometabolic diseases, but results may vary among different studies, and this study also reports certain threshold values, although their optimal thresholds still need to be identified in large-scale follow-up studies. After the adjustment for confounding factors, both the results and those of other studies indicate a positive association between VAI, LAP, and the risk of OSA. In subgroup analyses, this study has newly identified that higher levels of LAP and VAI are more likely to increase the odds of developing OSA in individuals younger than 60 years, while this association appears to diminish in those aged 60 years and above. This may be caused by age-related changes in metabolism and physiology, including alterations in lipid accumulation patterns and associated metabolic indicators [46, 47]. Elderly individuals are more susceptible to hypertension, diabetes, and cardiovascular diseases, which may influence sleep quality or metabolic indicators, thus attenuating the association between LAP, VAI, and OSA. Interestingly, Hai Deng and colleagues have also provided an explanation for this phenomenon, suggesting that this difference can be clarified by the contradictory impact of adipose tissue distribution on older adults [21]. Furthermore, Auyeung TW and his colleagues conducted a five-year longitudinal study involving 4000 elderly individuals, finding that older men exhibit resistance to the harmful effects of overweight and obesity; mild overweight or obesity may even have a protective effect [48].

### Study strengths and limitations

This research has certain advantages. As a vital epidemiological study in the United States, NHANES collects data with strict quality control procedures, and the conclusions drawn are quite reliable. The utilization of VAI and LAP enables a comprehensive evaluation of visceral fat distribution, devoid of the drawbacks of high costs, radiation exposure, and operational complexity associated with CT and MRI scans. As a result, this approach can be more widely used in clinical and screening settings. Additionally, this study’s results provide certain guiding significance for clinical practice. Clinical staff can better identify individuals with high VAI and LAP for OSA screening, as they can serve as very efficient diagnostic biomarkers. At the same time, clinicians can also develop more personalized treatment plans for patients based on VAI and LAP.

However, this study still has the following limitations: (1) The use of a cross-sectional design precludes the observation of dynamic changes in physical indicators. (2) Reliance on data from US adults may limit the generalizability to other populations. (3) Owing to the lack of polysomnography data collection in the NHANES process, OSA was determined based on NHANES questionnaire responses, which may introduce recall or self-report bias.

## Conclusion

In conclusion, this study utilizing NHANES data establishes a significant association between elevated LAP and VAI levels and the risk of OSA, suggesting their utility as predictive markers for OSA. Moderating LAP and VAI levels may not only delay OSA progression but also serve as preventive measures. Healthcare interventions focusing on dietary and exercise modifications are recommended for individuals with high LAP and VAI levels to mitigate OSA risk. The obtained findings underscore the importance of incorporating LAP and VAI assessments into clinical evaluations to guide targeted interventions and improve outcomes in individuals at risk of OSA.

## Data Availability

No datasets were generated or analysed during the current study.
